# Biomechanical Loads and Their Effects on Player Performance in NCAA D-I Male Basketball Games

**DOI:** 10.3389/fspor.2021.670018

**Published:** 2021-12-15

**Authors:** Sigrid B. H. Olthof, Tahmeed Tureen, Lam Tran, Benjamin Brennan, Blair Winograd, Ronald F. Zernicke

**Affiliations:** ^1^School of Sport and Exercise Sciences, Liverpool John Moores University, Liverpool, United Kingdom; ^2^Exercise and Sport Science Initiative, University of Michigan, Ann Arbor, MI, United States; ^3^Department of Biostatistics, University of Michigan, Ann Arbor, MI, United States; ^4^Michigan Institute for Data Science, University of Michigan, Ann Arbor, MI, United States; ^5^Department of Orthopedic Surgery, Michigan Medicine, University of Michigan, Ann Arbor, MI, United States; ^6^School of Kinesiology and Department of Biomedical Engineering, University of Michigan, Ann Arbor, MI, United States

**Keywords:** player tracking, mixed effects models, player performance, longitudinal analysis, performance analysis, periodization, accelerometer, team sports

## Abstract

Basketball games and training sessions are characterized by quick actions and many scoring attempts, which pose biomechanical loads on the bodies of the players. Inertial Measurement Units (IMUs) capture these biomechanical loads as PlayerLoad and Inertial Movement Analysis (IMA) and teams collect those data to monitor adaptations to training schedules. However, the association of biomechanical loads with game performance is a relatively unexplored area. The aims of the current study were to determine the statistical relations between biomechanical loads in games and training with game performance. Biomechanical training and game load measures and player-level and team-level game stats from one college basketball team of two seasons were included in the dataset. The training loads were obtained on the days before gameday. A three-step analysis pipeline modeled: (i) relations between team-level game stats and the win/loss probabilities of the team, (ii) associations between the player-level training and game loads and their game stats, and (iii) associations between player-level training loads and game loads. The results showed that offensive and defensive game stats increased the odds of winning, but several stats were subject to positional and individual performance variability. Further analyses, therefore, included total points [PTS], two-point field goals, and defensive rebounds (DEF REB) that were less subject to those influences. Increases in game loads were significantly associated with game stats. In addition, training loads significantly affected the game loads in the following game. In particular, increased loads 2 days before the game resulted in increased expected game loads. Those findings suggested that biomechanical loads were good predictors for game performance. Specifically, the game loads were good predictors for game stats, and training loads 2 days before gameday were good predictors for the expected game load. The current analyses accounted for the variation in loads of players and stats that enabled modeling the expected game performance for each individual. Coaches, trainers, and sports scientists can use these findings to further optimize training plans and possibly make in-game decisions for individual player performance.

## Introduction

Basketball is a high-demanding team sport that is characterized by many scoring attempts under the pressure of the shot clock. Consequently, players move quickly up and down the court, and their basketball-specific movements are characterized as accelerations, decelerations, changes of direction, and jumps (Petway et al., 2020a). Those movements put a biomechanical load on the body of each player (Vanrenterghem et al., 2017), are necessary to get a positional advantage over their opponent, and are essential for top-caliber basketball performance (Petway et al., 2020b). Basketball players need to be well prepared for high physical loads in combination with the dense schedule that is typical for the basketball competition. The dense schedule on the college basketball level requires players to compete in multiple games per week, with sometimes less than 3 days between games. Coaches, trainers, and sports scientists are therefore aiming for optimizing training strategies and periodization plans while accounting for the high demands of the basketball game (Conte et al., 2018a).

The development and implementation of wearable technology have enabled coaches, trainers, and sport scientists to monitor the on-court workload of basketball players (Russell et al., 2021). Players perform rapid movements with frequent changes of direction, which can be detected by Inertial Measurement Units (IMUs). IMUs containing accelerometers, gyroscopes, and magnetometers are able to detect movements and recognize accelerations, decelerations, changes of direction, or jumps of players. It is important to monitor the high physical demands of basketball to analyze the performance of players in the competition, understand the adaptations to the training program, and minimize the risks of overtraining and injuries (Aoki et al., 2017; Conte et al., 2018b). Various metrics have been proposed to capture the movements of basketball players as the external load during training and competition (Vanrenterghem et al., 2017; Russell et al., 2021). PlayerLoad and Inertial Movement Analysis (IMA) variables have been utilized to quantify those movements as biomechanical load, although the validity of many of these metrics requires further study (Russell et al., 2021). PlayerLoad is a validated variable in team sports to capture intermittent and multi-directional running (Barrett et al., 2016; Russell et al., 2021). IMA detects explosive movements above an acceleration threshold and captures these actions in a single direction. Although widely used by practitioners to monitor explosive basketball movements, IMA has not been validated yet (Russell et al., 2021).

College basketball teams compete in multiple games per week across a 5-month season (i.e., from November to the end of March/beginning of April). The National Collegiate Athletic Association (NCAA) organizes college basketball competition for university teams in different conferences and divisions. A conference in American collegiate athletics can be viewed as a subset league within the larger national league. Teams from the same conference compete for their respective conference championships and for the national title at the end of the season. Games in the competitive season are played against opponents from the same conference or cross-conference. The type of competition may not affect the biomechanical demands of games, with no differences in accelerations and decelerations between conference and non-conference games (Petway et al., 2020a). However, biomechanical game demands vary across positions, with each position (i.e., guards, forwards/wings, and centers/posts) demanding specific activities on the court (Vázquez-Guerrero et al., 2018). Coping with the high demands in competition, allowing recovery in between games, and preparing players for the next game to require a balanced and focused training program. A typical college basketball schedule with up to two or three games per week leaves little time between games to prepare for the next game. Generally, training demands are lower than game demands to facilitate this recovery and preparation (Petway et al., 2020b), but logically there is variation in the training load found for different drills (Svilar et al., 2019) and positions (Svilar et al., 2018).

Performance of basketball is measured by its game-related statistics to determine the efficiency of individual players and teams across the season (Sampaio et al., 2015). Stats of the players reflect their efficiency in scoring (i.e., two-point, three-point, and free throws), their contributions to the offense (i.e., assists and offensive rebounds [OFF REB]), and their contributions to defense (i.e., forced turnovers, blocks [BLK], steals [STL], and defensive rebounds [DEF REB]). Successful teams competing in NCAA Division-I (D-I) basketball reported higher percentages on both offensive and defensive metrics that includes three-points [PTS], free throws, and DEF REB (Conte et al., 2018a). Predominantly, situations where the National Basketball Association (NBA) players are in close proximity to the basket were associated with successful performance (Sampaio et al., 2015), such as two-point field goals and rebounds.

The collection of tracking data from IMUs and game statistics from box scores has led to an emerging volume and variety of data for team sports. Teams have integrated the use of Global or Local Positioning Systems (GPS and LPS, respectively), IMUs, and game stats during all phases of the season and have built multi-season databases. The accumulation of these data provides the foundation for artificial intelligence approaches, such as machine learning, to reveal patterns that could not be studied previously, with smaller datasets based on traditional observational analyses (Herold et al., 2019). Thus far, machine-learning methods have been used in team sports in a variety of sport performance applications, such as to study the outcome in basketball games (Zhang et al., 2020) and soccer matches (Leicht et al., 2017; Goes et al., 2019), advanced tactical analyses in soccer (Rein and Memmert, 2016; Lutz et al., 2020; Meerhoff et al., 2020), basketball plays (Wang et al., 2018; Chung, 2019), and to explore the reliability of game statistics (Franks et al., 2015; Pérez-Ferreirós et al., 2019) and IMU metrics (Rojas-Valverde et al., 2019). However, a relatively unexplored area is the relation between physical basketball demands and game performance. Vázquez-Guerrero et al. studied the correspondence between game performance and training loads in the 7 and 28 days leading up to the game (Vázquez-Guerrero et al., 2020). They, however, could not establish clear relations for positions, with the exception of an association between low-training workloads and good performance of a small forward. Given the many games played in only a few days, such as a shorter time window for the training period may be a more promising approach to find an effect on game performance (Oliveira et al., 2020).

Taken together, tracking data and game stats have afforded basketball coaches, trainers, and sport scientists valuable insights into the performance of their team. A holistic approach, i.e., both types of data, may lead to new insights about how biomechanical loads of a player relate to game-related statistics. Therefore, the purpose of the current study was to quantify successful basketball performance and understand the association between game and training loads and game performance. The first aim was to identify which team-level basketball metrics had significant effects on winning and losing. The second aim was to draw inferences on whether the biomechanical loads of the players during basketball games and training sessions had significant associations with game-related statistics of the players. The third aim was to draw inferences between training loads and game loads of the players. By connecting those aims, the current study provided new insights about how monitoring biomechanical loads during games and training sessions could better enable coaching and training staff to guide players to optimize their performance on the court.

## Materials and Methods

### Participants

Data from one collegiate male basketball team were collected during the 2016–17 and 2017–18 seasons. The team competed in a National College Athletic Association Division-I (NCAA D-I) Power Five conference, corresponding to a level 3 elite status (Russell et al., 2021). The students-athletes were recruited by the head coach of the university and were eligible to play at the collegiate level. Using the roster of the team, players were grouped as either guard, forward, or center players. Players provided informed consent prior to participation in data collection *via* the Department of Athletics at the University. Deidentified data from all players were compiled into a data repository, and the Institutional Review Board at the University approved secondary data analyses.

### Procedure

Player tracking device and box score data were collected from the 2016–17 to 2017–18 (Seasons 1 and 2, respectively) regular seasons and postseasons. Each player was assigned with an Optimeye S5 device (Catapult Innovations, Melbourne, Australia) to ensure consistency in the data collection. The Optimeye S5 device consisted of a 100 Hz tri-axial accelerometer, tri-axial gyroscope, and magnetometer and captured the multi-directional movements of the basketball players. The device was worn on the trunk between the shoulder blades at approximately the C7-T1 level and in a pouch attached to a team-issued shirt or in a harness. Devices were distributed prior to training and games. Data of inactivity before and after the activities were removed from further analyses.

Data were checked, cleaned, and organized in OpenField software (OpenField version 1.22.2; Catapult Innovations, Melbourne, Australia). Data collection was closely monitored during or directly after all activities and, for the purpose of the current study, the training and game data were viewed as entire sessions instead of a detailed analysis of training drills or game parts. Catapult data from training sessions and games were exported as .csv files for subsequent analysis in R.

Biomechanical load measures were of interest in the current study with the main variables being PlayerLoad and IMA. PlayerLoad was a vector variable calculated by the input of the tri-axial accelerometer. PlayerLoad reflects accumulated efforts and movements of a player on the court regardless of the body orientation or direction (in *arbitrary units*) of a player. IMA measures captured the accelerations of a player and corrected for orientation of a player using information from the tri-axial accelerometer and tri-axial gyroscope. It detected the body orientation of a player and counted the number of accelerations, decelerations, changes of direction, and jumps.

Game-related statistics were obtained at the team and player level from the box scores of the University Athletics Department. Offensive and defensive game statistics related to scoring at team level (total scores and percentages) and player level (total scores) consisted of field goals (made and attempted), three-point field goals (made and attempted), free throws (made and attempted), rebounds (offensive, defensive, and total), personal fouls, foul-outs, turnovers [DEF REB], BLK, and STL.

### Data Engineering

There were three original data sources used for the current study. The first dataset contained the biomechanical load measures with PlayerLoad and IMAs that were captured for 12 players per season during training sessions and games. The second dataset consisted of the game-by-game statistics with the game result of the team as wins/losses and team-level game stats. The third dataset consisted of player-level game stats from all players that made an appearance during a game. Due to players leaving and returning to the team after a season, the first dataset contained 16 unique players and the third dataset 23 unique players. Of note, each player from the first dataset occurred in the third dataset.

Players who did not participate in games for at least 10 mins *on average* were excluded from analyses since their individual game stats had a disproportionate amount of zero counts that were undesirable for analysis purposes (i.e., 0 PTS, 0 rebounds, and 0 STL). The training sessions were analyzed according to how many days away they were from gameday (i.e., gameday [GD] minus) (Malone et al., 2015). To illustrate, Training Load GD-1 refers to the load of a player observed 1 day before a game. To reduce the noise of possible games on the days leading up to gameday and to focus on the effect of training load on the game, observations were omitted that were outside the 2-day window span from gameday.

### Analytical Dataset and Sample

After relevant data processing and integration, the Season 1 dataset consisted of 722 observations, and the Season 2 dataset consisted of 785 observations. Players with an average game time of fewer than 10 mins were excluded from the dataset. In total, seven players (*n* = 2 guards, *n* = 2 forwards, and *n* = 3 centers) per season were part of the observations. Data were collected on the same players across a season-long time window providing a quasi-longitudinal study. Additionally, datasets had repeated measures for individuals since a player played more than one game per season. The datasets were reconstructed to analytical samples. In these samples, each row contained an observational sample with the game load and corresponding training loads of a player (i.e., the training loads 1 and 2 days before the game). The analytical sample of Season 1 consisted of 263 observations from seven regular players with 524 corresponding training observations. The analytical sample of Season 2 consisted of 278 observations from seven regular players with 528 corresponding training observations.

### Analysis Pipeline

**Figure 1:**
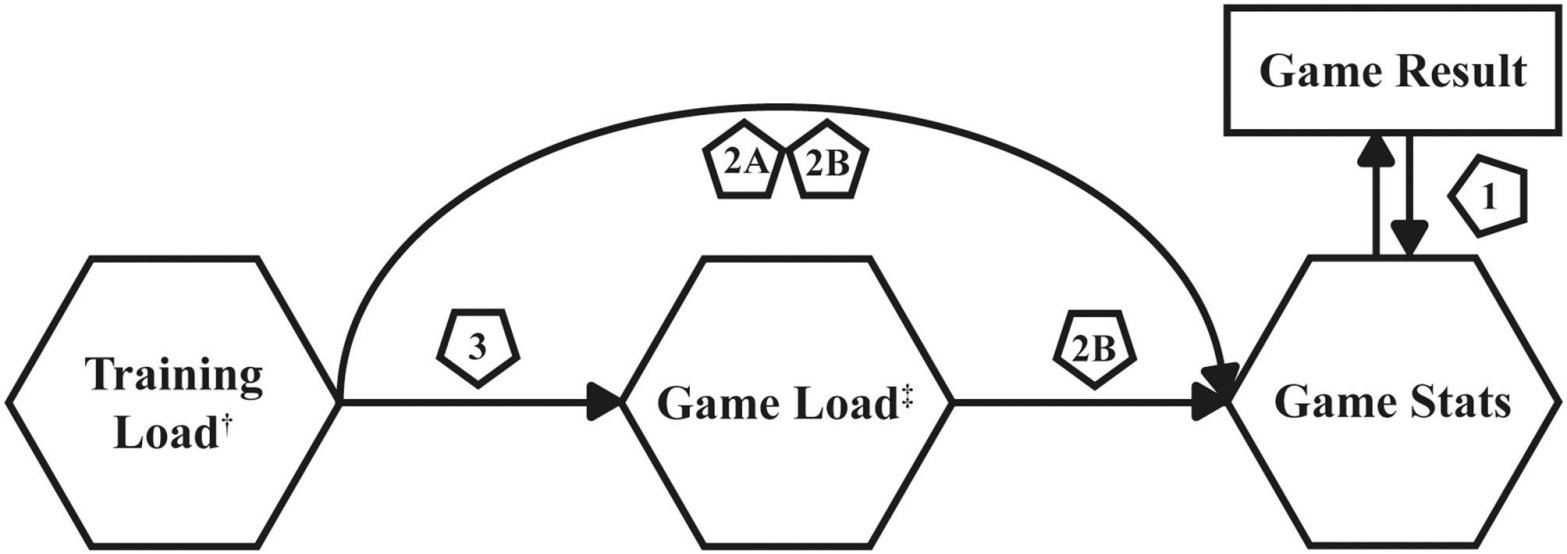
Process overview of the analysis pipeline. The pentagons contain numbers referring to the steps in the analysis pipeline. †: Along with other relevant game metrics, such as position and minutes played of a player and the venue and conference type of a game. ‡: Specific outcome variables deemed relevant by logistic regression analysis of game-level statistics and win/loss probabilities.

To address the research aims of the current study, a three-step analysis pipeline was used (Figure 1). In the first step of the analysis, logistic regression determined the statistical associations between team-level game stats and the win/loss probabilities of the team (Figure 1—label 1). That modeling approach allowed identification of which specific basketball metrics team stats had an effect on game results. The outcome of interest was an indicator of the team winning or losing the game. The predictors of interest were two-point shooting percentage (2FGP), three-point shooting percentage (3FGP), free throw percentage (FTP), OFF REB, DEF REB, TO, STL, and BLK at the team level. PTS were not included as a predictor as they are inherent to the purpose of the game: the team scoring the most PTS wins. Of note, that model was built using the team-level game stats (i.e., second dataset in 2.3. Data Engineering). Since there were fewer than 40 games in each season, both seasons were compiled in this dataset to run the analyses (*n* = 79 games), and no stratification by season was applied. To evaluate the statistical significance of the estimates of the odds ratios (predictor effects) from the logistic regression model, 95% Wald CIs were calculated, and their respective Wald test *p*-values (significance level: α = 0.05) were analyzed. CIs that did not include a value of 1 were considered as significant results. The mathematical illustration of that statistical model is provided in Supplementary Material Equation 1.

The second and third steps of the analysis included the biomechanical loads and player-level stats of a player. The game stats from step 1 that demonstrated significant associations with win-loss odds were used as outcomes of interest. PTS scored of a player were added to the game stats as an outcome, because scoring PTS is the most important aspect of winning games in basketball. Furthermore, game stats were dropped that were relatively rare and tended to vary substantially game-to-game. Hence, subsequent analyses included PTS scored, two-point field goals made (2FGM), and DEF RB. Lastly, IMA data were excluded from the predictors due to the high correlation between IMA and PlayerLoad (*r* = 0.91 and *r* = 0.89 for Seasons 1 and 2, respectively). IMA has not been validated yet as a metric to capture physical activity in team sports (Russell et al., 2021), it captures actions in a single direction, and it may potentially cancel out low-intensity movements when it is implementing an acceleration threshold of 1.5 m/s^2^ (as the default of the manufacturer). For those reasons, only PlayerLoad was included in further analyses.

In step two, the relations were modeled between player-level biomechanical loads of games and training sessions and player-level game stats. The predictors of interest were PlayerLoad during a game (Game Load), PlayerLoad during training 1 day (Training Load GD-1) and 2 days before the game (Training Load GD-2), conference game (yes/no), home game (yes/no), and player position (guard, forward, or center) of each player. The PlayerLoad predictors were divided by 100 for interpretability purposes in the models. The implicit assumption was made that players who played more minutes were innately able to score more PTS and get more rebounds. Therefore, an offset term was included in the models to account for the time a player played in each game.

Two types of models were fit for each player-level game stat: (i) training models, which included all the aforementioned predictors except Game Load (Figure 1—label 2a) and (ii) game models, which included all the aforementioned predictors (Figure 1—label 2b). The first model was used to calculate expected player performance *based solely* on training-level measures allowing coaches to get a better understanding of who was likely to score more PTS and rebounds before a game. The second model was used to calculate expected player performance during the game while monitoring changes in their game load. It modeled the effect of game load on game stats, controlling for the effects of training load, type of game, and player position. Since the player-level game stats were count measures, they were modeled using Poisson mixed-effects regressions with an offset term for minutes played. As noted previously, the analytical sample had repeated measures for the players since each player played multiple games throughout the season. Therefore, mixed-effects models were used to draw inferences on the associations between the player-level biomechanical load measures and their game stats. The guard is used as the reference position for modeling purposes. A random intercept was incorporated in the model to account for the repetition of the players in the data.

Finally, the model in the third step used Game Load as the outcome (Figure 1—label 3). Results from that model were used to assess how Training Load GD-1 and Training Load GD-2 may have affected Game Load of a player and whether there may have been a significant association between those measures. To model that outcome, a linear mixed-effects model was used, since the outcome was a continuous variable. That model included centered minutes played of each player (game minutes—season average game minutes) to account for the effects of greater on-court time, as offset terms were incompatible with linear models.

To evaluate the statistical significance of the estimates from those regression models, 95% Wald CIs were calculated and their respective Wald test *p*-values (significance level: α = 0.05) were observed. CIs that did not include a value of 1 were considered significant for the Poisson models and CIs that did not include a value of 0 were considered significant for the linear models. The mathematical illustration of those regression models in steps two and three are provided in Supplementary Material Equations 2 and 3, respectively. Those statistical analyses were performed using the *stats, lmerTest*, and *lme4* packages in R. The results were transformed into the odds ratio scale (from the log scale). Any estimate greater than 1.0 was considered as a positive effect on the outcome, and an estimate less than 1.0 was considered as a negative effect on the outcome.

## Results

Descriptive statistics from the analytical samples of both seasons are presented in Table 1. The results from step 1 of the analysis pipeline are summarized in Table 2. Results from the regression model indicated that a 1% increase in team 2FGP would have increased the odds of winning by 13% (100 × [1.13 – 1.00] = 13%) while holding other predictors constant. On the other hand, an increase of turnover count by one turnover would have decreased the expected winning odds of the team by 20% (100 × [0.80 – 1.00] = −20%) while holding other predictors constant. The model results suggested that 2FGP, FTP, DEF RB, STL, and BLK were statistically significant since their CIs did not cross the value of 1 and they increased the odds of winning. Additionally, after checking for variance inflation and statistical significance of the model results, the model predictors showed low correlation (*r* < 0.4 for non-time variables).

**Table 1:** Descriptive statistics of the analytical sample for Season 1 (*n* = 263) and Season 2 (*n* = 278). The mean (SD) is provided for continuous measures, and the count (percentage) is provided for categorical measures.

**Characteristic**	**Season 1**	**Season 2**
Guard	76 (28.9%)	80 (28.8%)
Forward	76 (28.9%)	81 (29.1%)
Center	111 (42.2%)	117 (42.1%)
Home games	138 (52.5%)	172 (61.9%)
Conference games	125 (47.5%)	120 (43.2%)
Game load	809.99 (257.50)	777.21 (272.47)
Training load GD-1	546.20 (224.96)	483.98 (162.91)
Training load GD-2	585.88 (388.97)	518.55 (268.07)
PTS	10.37 (6.40)	9.18 (6.82)
A	1.84 (2.30)	1.73 (1.94)
2FGM	2.32 (2.00)	2.30 (2.12)
3FGM	1.34 (1.30)	1.04 (1.28)
FTM	1.70 (2.13)	1.44 (1.78)
DEF RB	2.79 (2.15)	3.03 (2.38)
OFF RB	0.83 (1.14)	0.92 (1.16)
STL	0.74 (0.94)	0.77 (0.96)
BLK	0.37 (0.77)	0.38 (0.68)

*The counts represent the number of observations in the sample that belonged to a particular characteristic. GD, gameday; PTS, points scored; A, assists; 2FGM, 2-point field goals Made; 3FGM, 3-point field goals made; FTM, free throws made; DEF RB, defensive rebounds; OFF RB, offensive rebounds; STL, steals; BLK, blocks*.

**Table 2:** The estimates of the odds ratios [95% Wald CIs] were drawn from the logistic regression models applied to the team-level data (*n* = 79 games).

**Predictors**	**Odds ratios**
**Win-loss logistic regression**	
3-Point field goal percentage	1.02 [0.93, 1.11]
2-Point field goal percentage	1.13 [1.02, 1.28]*
Free throw percentage	1.08 [1.02, 1.15]*
Offensive rebounds	1.10 [0.83, 1.52]
Defensive rebounds	1.40 [1.17, 1.76]*
Turnovers	0.80 [0.64, 1.17]
Steals	1.64 [1.18, 2.59]*
Blocks	1.87 [1.20, 3.31]*

Bolded*: *results indicate a significant association (CI does not include 1)*.

For the second step of the analysis, free throws made (FTM), STL, and BLK were excluded as outcome variables in the modeling, because they occurred as relatively rare events and were subject to high amounts of game-to-game variance. To illustrate, there were 115 (43.7% of sample) zeros for FTM, 135 (51.3% of sample) for STL, and 196 (74.5% of sample) for BLK in Season 1, while in Season 2, there were 125 (45.0% of sample) zeros for FTM, 138 (49.6% of sample) for STL, and 199 (71.6% of sample) for BLK.

The results derived from the training and game models (steps 2A and 2B, respectively) are summarized in Table 3 and stratified per season. The training and game load sorted different effects on the game stats in both seasons, highlighting the seasonal effects. Notably, Training Load GD-2 had a significant negative effect on the DEF RB of a player in Season 1. For the same season, Game Load had significant positive effects on PTS and 2FGM of a player. Results from Season 2 showed that Training Load GD-2 had a positive effect on a PTS and 2FGM of a player. Taking the guard as the reference position, a 100-unit increase in Training Load GD-2 would have increased their expected PTS by 3% and 2FGM by 8% for that respective game while holding other predictors constant. The Game Model from Season 2 suggested a similar relation between Training Load GD-2 and the respective offensive game stats. Moreover, that model also suggested that an increase in Game Load could increase their DEF RB. Lastly, the center position positively impacted the game results in both seasons, and there was a significant influence of home and conference games on the game stats in season 2.

**Table 3:** The estimates of the multiplicative effects [95% Wald CIs] were drawn from the Poisson mixed-effects regression models applied to the analytical sample from Season 1 (*n* = 263).

**Predictors**	**PTS**	**2FGM**	**DEF RB**
**Season 1 training model**			
**Guard (ref.)**			
Forward	1.03 [0.74, 1.43]	1.25 [0.78, 1.99]	0.99 [0.64, 1.53]
Center	1.08 [0.80, 1.46]	1.82 [1.20, 2.77]*	1.21 [0.81, 1.80]
Training load GD-1	1.00 [0.99, 1.02]	1.00 [0.97, 1.03]	1.00 [0.98, 1.03]
Training load GD-2	0.99 [0.99, 1.00]	0.99 [0.97, 1.01]	0.98 [0.96, 1.00]*
Home	1.08 [1.00, 1.17]	1.05 [0.89, 1.24]	0.96 [0.83, 1.12]
Conference	0.95 [0.88, 1.03]	0.96 [0.81, 1.14]	0.88 [0.75, 1.03]
**Season 1 Game Model**			
**Guard (ref.)**			
Forward	0.89 [0.78, 1.47]	1.36 [0.91, 2.02]	1.00 [0.67, 1.51]
Center	1.14 [0.85, 1.53]	2.05 [1.43, 2.95]*	1.23 [0.85, 1.80]
Training load GD-1	1.01 [0.99, 1.03]	1.01 [0.98, 1.05]	1.01 [0.98, 1.04]
Training load GD-2	0.99 [0.98, 1.00]	0.98 [0.96, 1.00]	0.98 [0.96, 1.00]
Game load	1.03 [1.01, 1.05]*	1.08 [1.03, 1.12]*	1.01 [0.97, 1.05]
Home	1.05 [0.97, 1.13]	1.04 [0.88, 1.22]	0.96 [0.82, 1.11]
Conference	0.94 [0.87, 1.01]	0.93 [0.78, 1.09]	0.87 [0.75, 1.02]
**Season 2 training model**			
**Guard (ref.)**			
Forward	1.21 [0.71, 2.05]	0.98 [0.43, 2.21]	1.04 [0.62, 1.75]
Center	0.99 [0.61, 1.61]	1.18 [0.56, 2.49]	1.47 [0.91, 2.36]
Training load GD-1	0.99 [0.97, 1.01]	1.00 [0.96, 1.05]	0.98 [0.95, 1.02]
Training load GD-2	1.03 [1.01, 1.04]*	1.04 [1.02, 1.07]*	1.00 [0.98, 1.03]
Home	0.95 [0.87, 1.03]	1.01 [0.85, 1.19]	1.20 [1.03, 1.39]*
Conference	0.88 [0.81, 0.96]*	0.80 [0.68, 0.94]*	0.95 [0.83, 1.10]
**Season 2 game model**			
**Guard (ref.)**			
Forward	1.22 [0.73, 2.04]	0.99 [0.45, 2.18]	1.07 [0.67, 1.71]
Center	1.01 [0.63, 1.63]	1.22 [0.59, 2.53]	1.58 [1.02, 2.45]*
Training load GD-1	0.99 [0.97, 1.01]	1.00 [0.96, 1.05]	0.98 [0.94, 1.02]
Training load GD-2	1.03 [1.01, 1.04]*	1.04 [1.01, 1.07]*	1.00 [0.98, 1.02]
Game load	1.01 [0.99, 1.03]	1.01 [0.98, 1.05]	1.03 [1.00, 1.06]*
Home	0.96 [0.88, 1.04]	1.02 [0.86, 1.20]	1.21 [1.05, 1.41]*
Conference	0.89 [0.82, 0.96]*	0.80 [0.68, 0.94]*	0.96 [0.83. 1.11]

Bolded*: *results indicate a significant association (CI does not include 1). GD, gameday; PTS, points scored; 2FGM, 2-point field goal made; DEF RB, defensive rebound*.

Findings from step 3 for the effects of training load on Game Load are summarized in Table 4. For Season 2, higher levels of Training Load GD-1 were observed to have a negative effect on Game Load. The analysis suggested that a 100-unit increase during training 1 day before a game would decrease Game Load of a players by 20.21 units while holding other variables constant. On the other hand, a 100 unit increase during training 2 days before a game would lead to an expected increase of Game Load of a player by 11.01 units. The load predictors were scaled by 100 units. Therefore, the predictor effects are presented for 100 increment interpretations. As for Season 2, there was not enough evidence to suggest a significant association between training load of a player 1 day before a game and Game Loads. Nonetheless, the model was able to detect a significant association between training load of players 2 days before a game and their game load.

**Table 4:** The estimates of the multiplicative effects [95% Wald CIs] drawn from the linear mixed-effects regression models applied to the analytical samples from Season 1 (*n* = 263) and Season 2 (*n* = 278) for modeling gameday player loads.

**Predictors**	**Game load season 1**	**Game load season 2**
**Game load model**		
**Guard (ref.)**		
Forward	−120.84 [−473.86, 232.17]	−40.00 [−275.24, 195.24]
Center	−183.23 [−505.53, 139.06]	–224.87 [–439.74, –10.00]*
Training load GD-1	–20.21 [–28.42, –11.70]*	1.83 [−10.11, 13.86]
Training load GD-2	11.01 [5.76, 16.22]*	10.77 [2.49, 18.84]*
Home	47.93 [5.81, 89.66]*	−48.44 [−99.77, 3.13]
Conference	38.86 [−3.48, 80.88]	−40.64 [−93.72, 12.69]
Centered minutes played	16.13 [12.60, 19.67]*	15.27 [11.38, 19.17]*

Bolded*: *results indicate a significant association (CI does not include 1). GD, gameday*.

It is important to note that although the model results suggest linear relations, the model does not imply an infinite increase or decrease in predictor values. The predictor values are limited to their minimum and maximum values observed in the dataset. Thus, the conclusions drawn here do not suggest that players can continue to increase their load without a limit (i.e., fatigue).

## Discussion

The current study determined statistical relations between biomechanical loads in games and training and basketball game performance in a collegiate male basketball team. There were three main findings related to the game result, game stats, and game load. Several offensive and defensive game stats were related to the game result. In addition, training load of the player 2 days before the game and the game load had significant effects on the points scored, two-point field goals, and defensive rebounds across two seasons. Finally, training load, the venue, and the minutes played of the player had a significant effect on the game load.

Consistent with the purpose of a basketball game, the findings of the current study showed that positive offensive and defensive stats increased the odds of winning the game. The results showed that higher percentages in two-point field goals and free throws and more defensive rebounds and blocks increased the win probabilities in collegiate basketball. The finding that a better performance in shooting and rebounding increased the chances of winning aligned with a previous study in NCAA D-I male basketball (Conte et al., 2018a). However, turnovers, steals, blocks, and rebound stats showed large to very large CIs for the odds ratios. That behavior suggested uncertainty in the odds ratio estimation even though the result was statistically significant. Because many game outcomes were highly dependent on position and individual performance, some measures were zero values even though the game stats from two seasons were compiled into one dataset. That could detract from the model-based inferential analysis. The game stats that remained in the current analysis aligned with previous observations that defensive rebounds and two-point field goals contribute to game performance (Sampaio et al., 2015). Alternatively, a combined overall player performance metric may be used for future studies to represent the game performance and include the omitted game stats (Vázquez-Guerrero et al., 2020), such as Performance Index Rating used by European basketball leagues or the NBA's Efficiency rating or Player Efficiency Rating.

The training and game models revealed significant associations between biomechanical load and expected game stats. The training model was designed to allow predictions based solely on the training loads on the 2 days leading up to gameday. The findings indicated that especially higher training loads 2 days before the game were associated with game performance, but also revealed different relations with offensive and defensive game stats across seasons. Those seemingly contradictory findings appeared by stratifying the analysis by season, but highlighted the association between training load and game performance. Whereas Vázquez-Guerrero et al. did not find correspondence between game performance and training load over longer periods (Vázquez-Guerrero et al., 2020), the current study included the training loads from a short time window of only 2 days and revealed significant associations with game performance. Apparently, although acknowledging that conditioning in team sports is a long-term process (Aoki et al., 2017), variations in training loads affected the performance in games in the short term.

Game loads were included in the game model to determine inferential relations between training and game loads with game stats. Higher than average game loads positively influenced the game stats, but demonstrated different effects in both seasons. Higher success rates in shooting and defensive actions elicited higher physical demands in the game. In an earlier description of game performance profiles, it was suggested that high performance in defensive tasks in the game was related to proficient fitness levels of basketball players (Sampaio et al., 2015). A causal relation between game loads and game stats, however, was difficult to draw due to the different constructs of game stats and biomechanical load. Perhaps players with good defensive skills possessed good physical capacities or that the defensive skills required players to give extra effort to be successful. Either way, with the advancements of technology and the means to monitor the physical demands in real-time during the game, the game model could enable coaches and sport scientists to make in-game decisions based on the physical demands during a game and the expected effect on the game stats.

Along with the relations among biomechanical training and game loads and game stats, game minutes, venue, and conference type were also significantly associated with game performance. To illustrate, the centered minutes played had a positive impact on the game load across two seasons. That result highlighted that with every minute played more than average, the expected biomechanical load in the game increased. That was an obvious result, as players needed to be on the court to be able to shoot and score. Given that per season seven players were included who averaged 10 minutes or more per game, only a small group of players were on the court for the majority of the game time during the season. Most likely, those players were well-trained by the games they consecutively played (Sampaio et al., 2015). This association of playing minutes, together with playing home or away and playing against conference opponents, highlights the importance of including contextual factors to model the effect on game performance.

The final model showed the relation between the training load and the load experienced during the game. Again, that model was capable of demonstrating the significant effects of training load on the game load, but seasonal differences appeared. In more detail, a higher training load than normal 1 day before the game reduced the expected game load in Season 1, but a higher training load than normal 2 days before the game was followed up by higher game loads in both seasons. Those findings may be exemplary for periodization plans despite the irregular schedule of the basketball competition that induced weekly variations (Conte et al., 2018a). The consistent finding across two seasons of the positive effects of training load indicated that a coach could look for opportunities in the competition schedule to work harder than average 2 days before a game and work lighter than average 1 day before a game. However, as multiple games are played during the season, that should be done with caution to allow for sufficient recovery for the high intensities demanded by the game. For example, the training session 2 days before a game could simultaneously be the training session the day after a previous game. In that case, a coach would likely use that training as a recovery session rather than overload players with another physical stimulus. It is therefore important to model external loads against their baseline and account for the physical variability of individuals across the season.

Although the current study did not include a prediction-based analysis, the analysis pipeline accounted for the individual variability between players. That variability may include psychological factors and individual performance differences. At the top level of NBA basketball, game performance is different for all-star players vs. non-all-star players (Sampaio et al., 2015) and arguably even within this select group of all-star players. In the current study, an assumption was that each player in the study had individual game performance profiles (i.e., so-called three-point shooters, clutch shooters, and big men). When that individual-level variability is not taken into account, analyses make the naive assumption that every player is expected to have the same baseline (or starting point). To illustrate, consider a starter and a non-starter with similar training loads. If the analysis does not account for individual variability, then they would have had similar expected scoring capabilities. However, once the individual variability was accounted for, the starter would have a higher expected scoring capability, because their baseline (training loads at zero) would be higher than the non-starter's baseline. Therefore, applying mixed models to sports analytics—especially studies that track multiple players over a period of time—may be beneficial since their measures are re*-*observed multiple times. Measures from the same players are inherently correlated with each other, because they were coming from the same player over a period of time.

In conclusion, the current study used mixed-models analysis to draw inferential relations between players' biomechanical loads in basketball games and training sessions with basketball performance in games from one collegiate basketball team. Although observations from one team with a relatively small number of players may limit the impact of the analysis, it should be noted that this is the first study using two seasons of data from the competitive season with player-level data from games and the two training sessions prior to the game. Several stats related to scoring and offensive and defensive contributions increased the odds of winning. However, most game stats were disproportionately distributed among the players, causing high uncertainty for the odds ratio estimation, and were therefore omitted from further analysis. In addition, biomechanical loads in training sessions and in the game were significantly associated with game stats. Current results showed significant associations between training loads and game load as well as between game loads and game stats. Training loads were good predictors for game loads, probably because they both reflected the player's biomechanical performance. Game loads were good predictors for game stats, likely because they were observed on the same activity. However, although the models were capable of demonstrating a relation of biomechanical loads with game performance, it is important to address the season-to-season variation that may have occurred by changes in the team roster, injuries, or competition schedule. Despite that seasonal variation, the biomechanical load 2 days prior to the game seemed to have a positive association with the game load and the game stats. Coaches, trainers, and sport scientists may find the current findings useful for optimizing training plans and potentially making in-game decisions for individual player performance.

## Data Availability Statement

The data analyzed in this study is subject to the following licenses/restrictions: The data were compiled into a data repository by the University's Department of Athletics. Requests to access these datasets should be directed to lisaraba@umich.edu.

## Ethics Statement

The studies involving human participants were reviewed and approved by Institutional Review Board University of Michigan. The patients/participants provided their written informed consent to participate in this study.

## Author Contributions

SO, BW, and RZ conceptualized the study. TT, LT, and BB organized the database and performed the statistical analyses. SO contributed to the original manuscript draft. TT was a contributing author on the manuscript. All authors made substantial contributions to the manuscript and reviewed and approved the submitted version.

## Conflict of Interest

The authors declare that the research was conducted in the absence of any commercial or financial relationships that could be construed as a potential conflict of interest.

## Publisher's Note

All claims expressed in this article are solely those of the authors and do not necessarily represent those of their affiliated organizations, or those of the publisher, the editors and the reviewers. Any product that may be evaluated in this article, or claim that may be made by its manufacturer, is not guaranteed or endorsed by the publisher.
