# Supplementary material for: Biomechanical Loads and Their Effects on Player Performance in NCAA D-I Male Basketball Games
Source: Front Sports Act Living. 2021 Dec 15;3:670018. doi: 10.3389/fspor.2021.670018 (PMC8714934; doi:10.3389/fspor.2021.670018)
Supplement: Supplementary file 1 [file Data_Sheet_1.docx]

***Supplementary Material***

**Equation S1**

$$\log\left[ \frac{P\left( Y_{i}=1|X \right)}{1-\left[ P\left( Y_{i}=1|X \right) \right]} \right]=\beta_{0}+\beta_{1}{2FGP}_{i}+\beta_{2}{3FGP}_{i}+\beta_{3}{FTP}_{i}+\ldots+\beta_{8}{BLK}_{i}$$

- i: i-th game
- Y: Win (Y = 1) or Loss (Y = 0)
- X: Matrix consisting of information regarding the predictor variables
- 2FGP: 2-Point Field Goal Percentage
- 3FGP: 3-Point Field Goal Percentage
- FTP: Free Throw Percentage
- …: Rest of the predictors described in 2.5 Analysis Pipeline
- BLK: Blocks
- β*_p_*: Log odds ratio of the p-th predictor
- exp(β*_p_*): Odds ratio of the p-th predictor (predictor effect on win/loss probability)

**Equation S2**

$$\log\left[ X_{j};b_{0i} \right]=b_{0i}+\beta_{0}+\beta_{1}{POS}_{i}+\beta_{2}{GL}_{ij}+\beta_{3}{TL1}_{ij}+\beta_{4}{TL2}_{ij}+\ldots+ \log({MIN}_{ij})$$

- i: i-th player
- j: j-th game
- Y_ij_: Game Stats (PTS/2FGM/DEF RB by player i in game j)
- X_j_: Matrix consisting of information regarding the predictor variables for game j
- b_0i_: Random intercept for i-th individual
- GL_ij_: Game Load for i-th individual in game j; only in game models
- TL1_ij_: Training Load one day before game for i-th individual in game j
- TL2_ij_: Training load two days before game for i-th individual in game j
- …: CONFG_j_ (conference: CONFG = 1 or non-conference: CONFG = 0) and HOMEG_j_ (home game: HOMEG = 1 or away game: HOMEG = 0)
- MIN_ij_: minutes played by the i-th individual in game j
- $\log\left[ X_{j};b_{0i} \right]$: equivalent to E[Y_ij_]
- exp(p): Rate ratio of the p-th predictor (predictor effect on win/loss probability)

**Equation S3**

$$Y_{IJ}|X_{J};b_{01}=b_{0i}+\beta_{0}+\beta_{1}{POS}_{i}+\beta_{2}{TL1}_{ij}+\beta_{3}{TL2}_{ij}+\ldots+ \beta_{6}{CMIN}_{ij}$$

- i: i-th player
- j: j-th game
- Y_ij_: Continuous outcome (Game Load by player i in game j)
- X_j_: Matrix consisting of information regarding the predictor variables for game j
- b_0i_: Random intercept for i-th individual
- PL1_ij_: Training load one day before game for i-th individual in game j
- PL2_ij_: Training load two days before game for i-th individual in game j
- …: CONFG_j_ (conference: CONFG = 1 or non-conference: CONFG = 0) and HOMEG_j_ (home game: HOMEG = 1 or away game: HOMEG = 0)
- CMIN_ij_: centered minutes for i-th individual in game j (game minutes in game j - season average game minutes)
